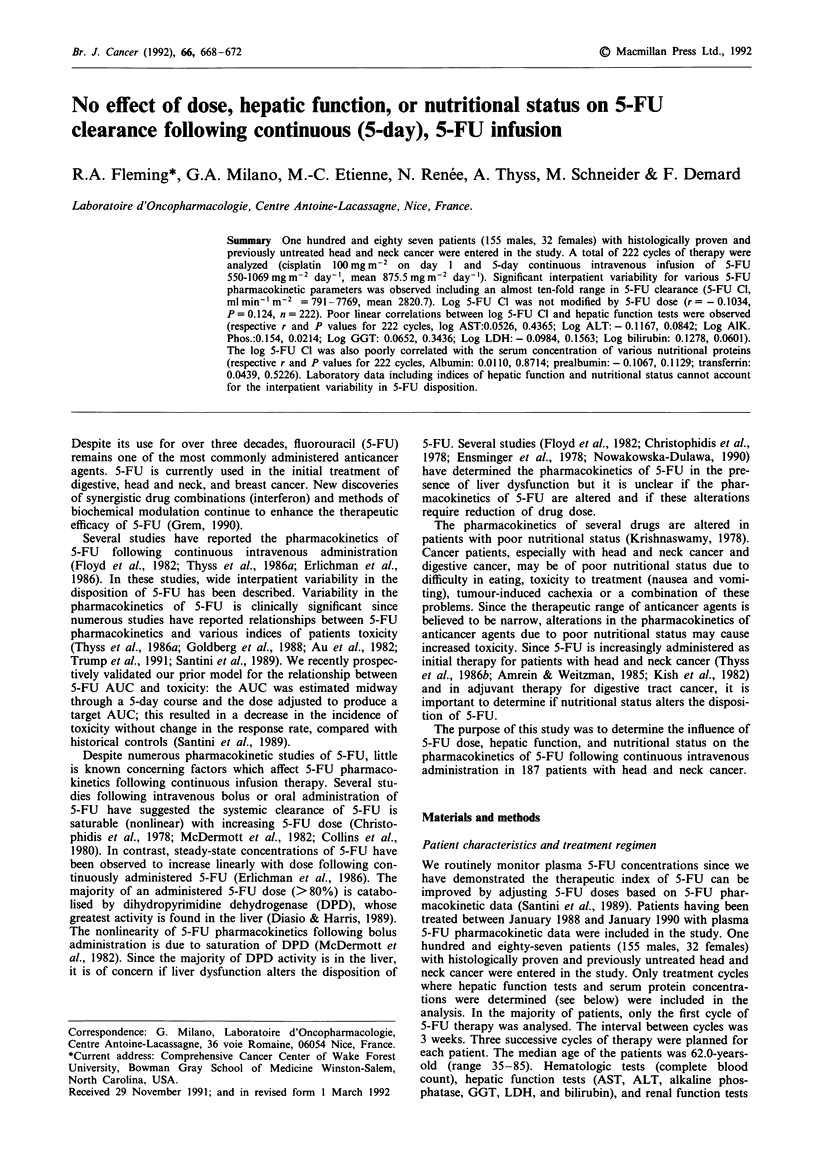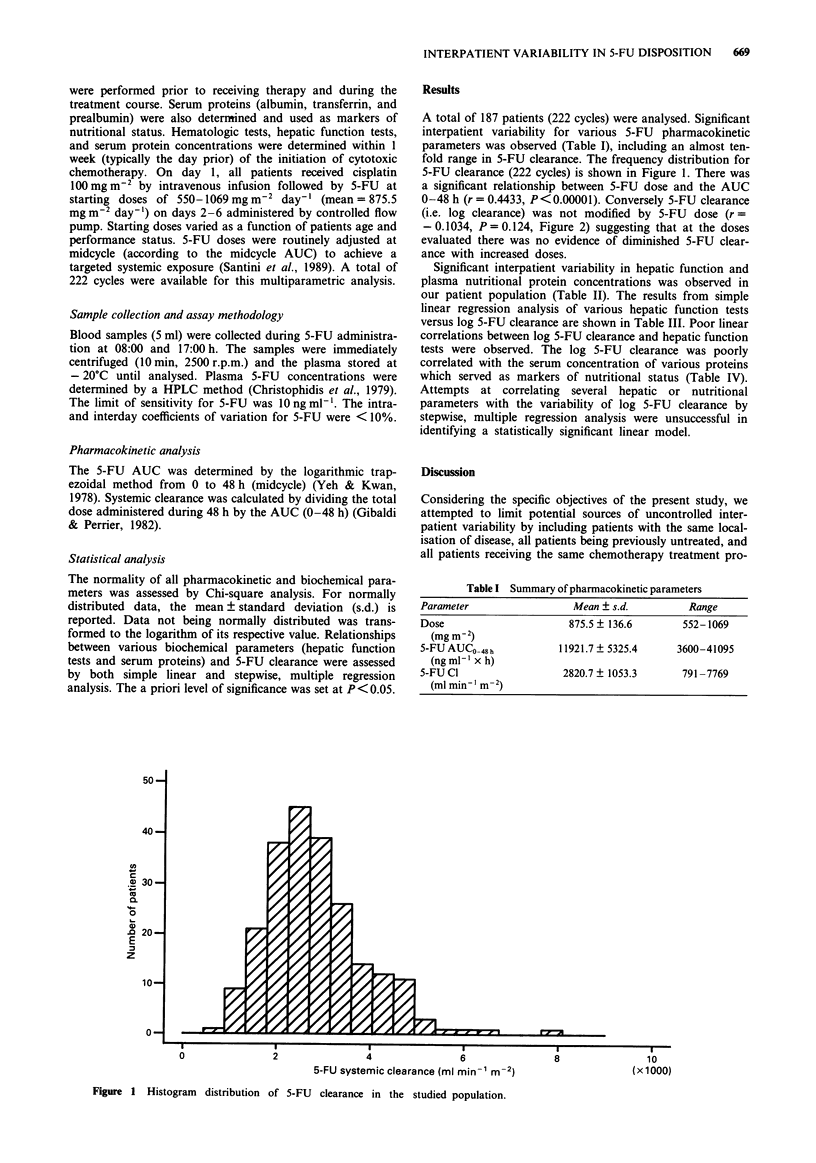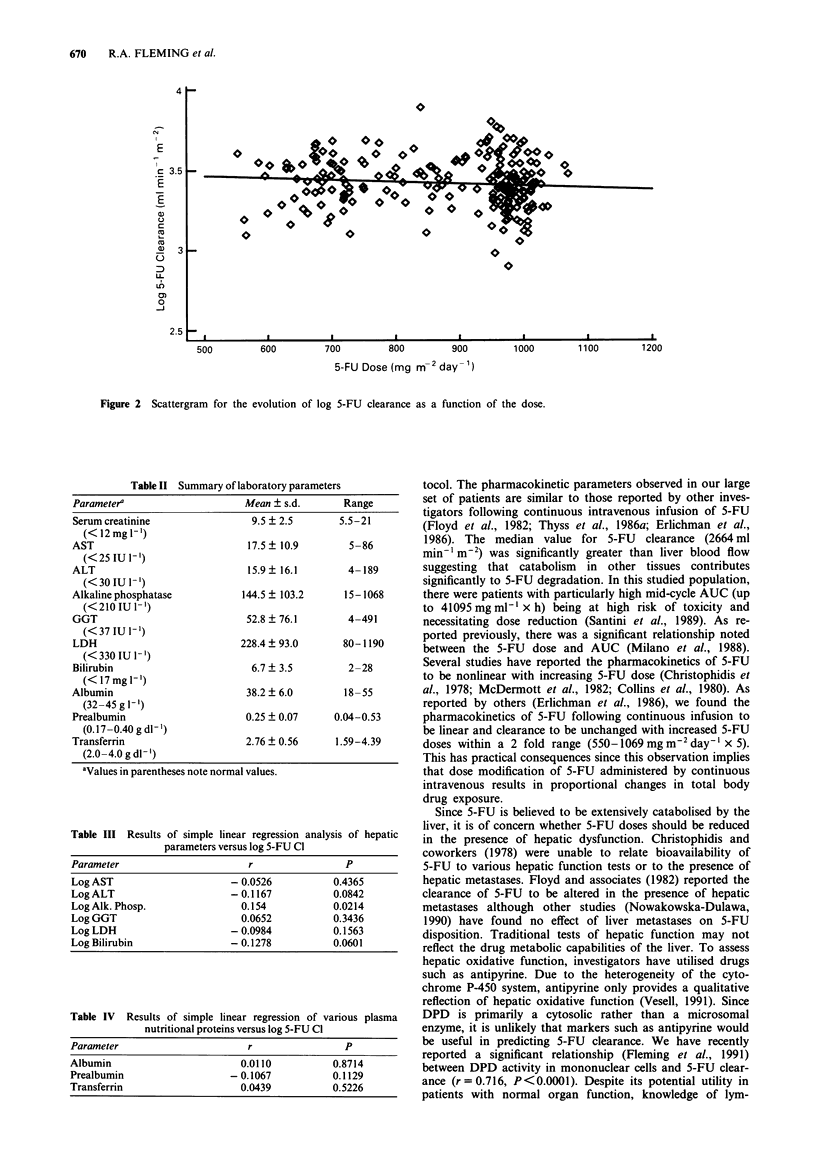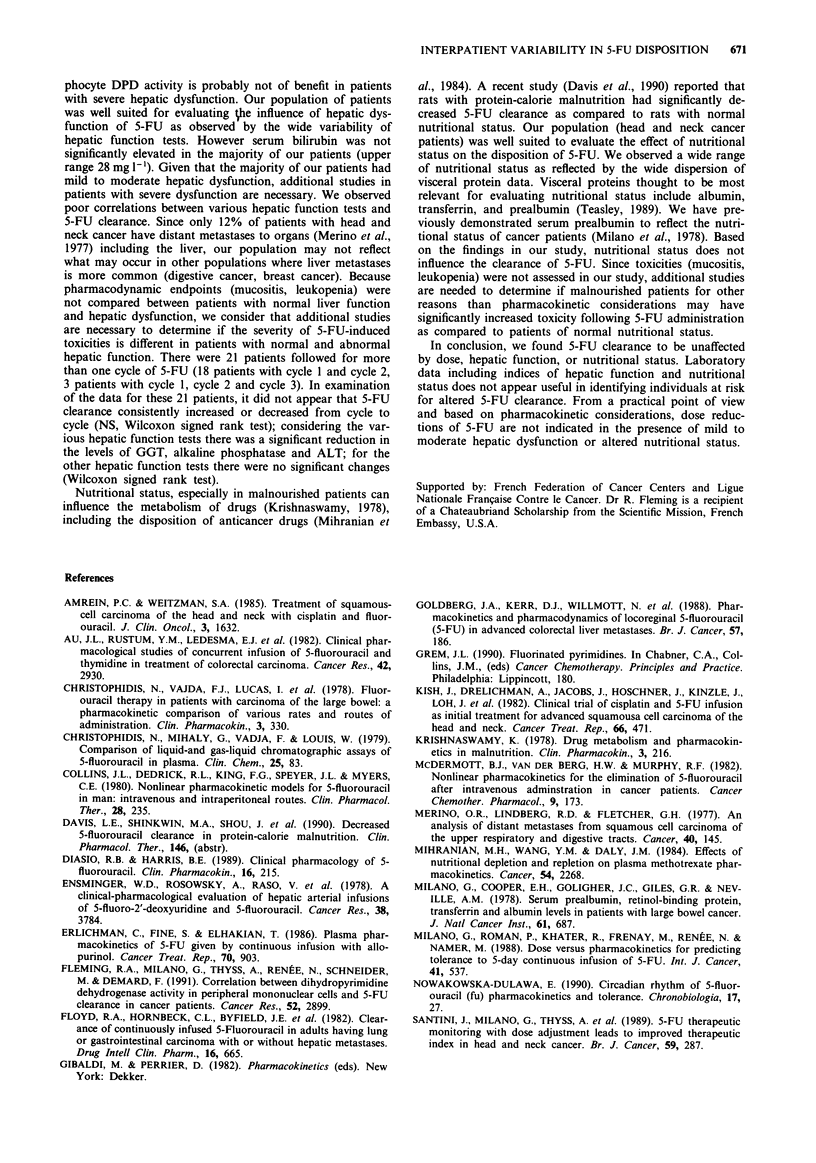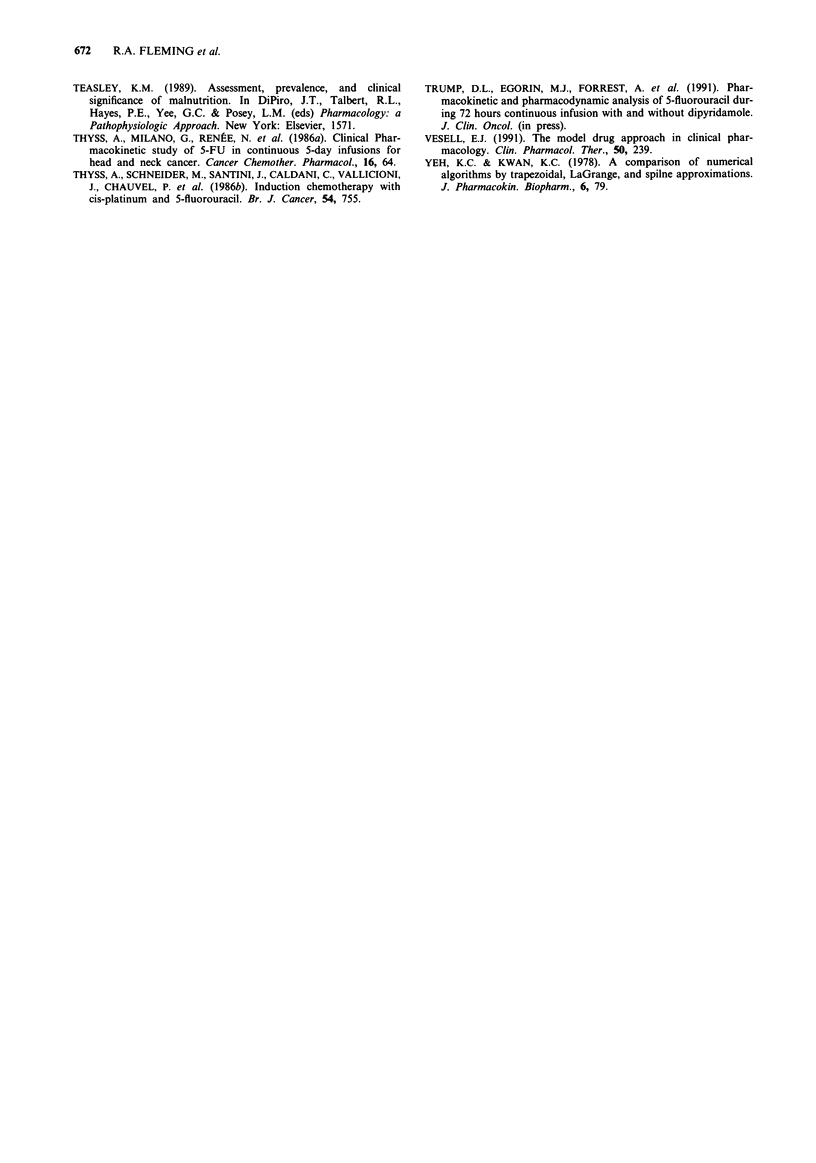# No effect of dose, hepatic function, or nutritional status on 5-FU clearance following continuous (5-day), 5-FU infusion.

**DOI:** 10.1038/bjc.1992.335

**Published:** 1992-10

**Authors:** R. A. Fleming, G. A. Milano, M. C. Etienne, N. Renée, A. Thyss, M. Schneider, F. Demard

**Affiliations:** Laboratoire d'Oncopharmacologie, Centre Antoine-Lacassagne, Nice, France.

## Abstract

One hundred and eighty seven patients (155 males, 32 females) with histologically proven and previously untreated head and neck cancer were entered in the study. A total of 222 cycles of therapy were analyzed (cisplatin 100 mg m-2 on day 1 and 5-day continuous intravenous infusion of 5-FU 550-1069 mg m-2 day-1, mean 875.5 mg m-2 day-1). Significant interpatient variability for various 5-FU pharmacokinetic parameters was observed including an almost ten-fold range in 5-FU clearance (5-FU Cl, ml min-1 m-2 = 791-7769, mean 2820.7). Log 5-FU Cl was not modified by 5-FU dose (r = -0.1034, P = 0.124, n = 222). Poor linear correlations between log 5-FU Cl and hepatic function tests were observed (respective r and P values for 222 cycles, log AST:0.0526, 0.4365; Log ALT: -0.1167, 0.0842; Log A1K. Phos.:0.154, 0.0214; Log GGT: 0.0652, 0.3436; Log LDH: -0.0984, 0.1563; Log bilirubin: 0.1278, 0.0601). The log 5-FU Cl was also poorly correlated with the serum concentration of various nutritional proteins (respective r and P values for 222 cycles, Albumin: 0.0110, 0.8714; prealbumin: -0.1067, 0.1129; transferrin: 0.0439, 0.5226). Laboratory data including indices of hepatic function and nutritional status cannot account for the interpatient variability in 5-FU disposition.


Br. J. Cancer (1992), 66, 668-672 © Macmillan Press Ltd., 1992

### No effect of dose, hepatic function, or nutritional status on 5-FU clearance following continuous (5-day), 5-FU infusion

R.A. Fleming*, G.A. Milano, M.-C. Etienne, N. Renée, A. Thyss, M. Schneider & F. Demard

*Laboratoire d'Oncopharmacologie, Centre Antoine-Lacassagne, Nice, France.*

**Summary** One hundred and eighty seven patients (155 males, 32 females) with histologically proven and previously untreated head and neck cancer were entered in the study. A total of 222 cycles of therapy were analyzed (cisplatin 100 mg $m^{-2}$ on day 1 and 5-day continuous intravenous infusion of 5-FU 550-1069 mg $m^{-2}$ $day^{-1}$, mean 875.5 mg $m^{-2}$ $day^{-1}$). Significant interpatient variability for various 5-FU pharmacokinetic parameters was observed including an almost ten-fold range in 5-FU clearance (5-FU Cl, ml $min^{-1}$ $m^{-2}$ = 791–7769, mean 2820.7). Log 5-FU Cl was not modified by 5-FU dose ($r = -0.1034$, $P = 0.124$, $n = 222$). Poor linear correlations between log 5-FU Cl and hepatic function tests were observed (respective $r$ and $P$ values for 222 cycles, log AST:0.0526, 0.4365; Log ALT: – 0.1167, 0.0842; Log AlK. Phos.:0.154, 0.0214; Log GGT: 0.0652, 0.3436; Log LDH: – 0.0984, 0.1563; Log bilirubin: 0.1278, 0.0601). The log 5-FU Cl was also poorly correlated with the serum concentration of various nutritional proteins (respective $r$ and $P$ values for 222 cycles, Albumin: 0.0110, 0.8714; prealbumin: – 0.1067, 0.1129; transferrin: 0.0439, 0.5226). Laboratory data including indices of hepatic function and nutritional status cannot account for the interpatient variability in 5-FU disposition.

Despite its use for over three decades, fluorouracil (5-FU) remains one of the most commonly administered anticancer agents. 5-FU is currently used in the initial treatment of digestive, head and neck, and breast cancer. New discoveries of synergistic drug combinations (interferon) and methods of biochemical modulation continue to enhance the therapeutic efficacy of 5-FU (Grem, 1990).

Several studies have reported the pharmacokinetics of 5-FU following continuous intravenous administration (Floyd *et al.*, 1982; Thyss *et al.*, 1986*a*; Erlichman *et al.*, 1986). In these studies, wide interpatient variability in the disposition of 5-FU has been described. Variability in the pharmacokinetics of 5-FU is clinically significant since numerous studies have reported relationships between 5-FU pharmacokinetics and various indices of patients toxicity (Thyss *et al.*, 1986*a*; Goldberg *et al.*, 1988; Au *et al.*, 1982; Trump *et al.*, 1991; Santini *et al.*, 1989). We recently prospectively validated our prior model for the relationship between 5-FU AUC and toxicity: the AUC was estimated midway through a 5-day course and the dose adjusted to produce a target AUC; this resulted in a decrease in the incidence of toxicity without change in the response rate, compared with historical controls (Santini *et al.*, 1989).

Despite numerous pharmacokinetic studies of 5-FU, little is known concerning factors which affect 5-FU pharmacokinetics following continuous infusion therapy. Several studies following intravenous bolus or oral administration of 5-FU have suggested the systemic clearance of 5-FU is saturable (nonlinear) with increasing 5-FU dose (Christophidis *et al.*, 1978; McDermott *et al.*, 1982; Collins *et al.*, 1980). In contrast, steady-state concentrations of 5-FU have been observed to increase linearly with dose following continuously administered 5-FU (Erlichman *et al.*, 1986). The majority of an administered 5-FU dose (>80%) is catabolised by dihydropyrimidine dehydrogenase (DPD), whose greatest activity is found in the liver (Diasio & Harris, 1989). The nonlinearity of 5-FU pharmacokinetics following bolus administration is due to saturation of DPD (McDermott *et al.*, 1982). Since the majority of DPD activity is in the liver, it is of concern if liver dysfunction alters the disposition of 5-FU. Several studies (Floyd *et al.*, 1982; Christophidis *et al.*, 1978; Ensminger *et al.*, 1978; Nowakowska-Dulawa, 1990) have determined the pharmacokinetics of 5-FU in the presence of liver dysfunction but it is unclear if the pharmacokinetics of 5-FU are altered and if these alterations require reduction of drug dose.

The pharmacokinetics of several drugs are altered in patients with poor nutritional status (Krishnaswamy, 1978). Cancer patients, especially with head and neck cancer and digestive cancer, may be of poor nutritional status due to difficulty in eating, toxicity to treatment (nausea and vomiting), tumour-induced cachexia or a combination of these problems. Since the therapeutic range of anticancer agents is believed to be narrow, alterations in the pharmacokinetics of anticancer agents due to poor nutritional status may cause increased toxicity. Since 5-FU is increasingly administered as initial therapy for patients with head and neck cancer (Thyss *et al.*, 1986*b*; Amrein & Weitzman, 1985; Kish *et al.*, 1982) and in adjuvant therapy for digestive tract cancer, it is important to determine if nutritional status alters the disposition of 5-FU.

The purpose of this study was to determine the influence of 5-FU dose, hepatic function, and nutritional status on the pharmacokinetics of 5-FU following continuous intravenous administration in 187 patients with head and neck cancer.

### Materials and methods

### *Patient characteristics and treatment regimen*

We routinely monitor plasma 5-FU concentrations since we have demonstrated the therapeutic index of 5-FU can be improved by adjusting 5-FU doses based on 5-FU pharmacokinetic data (Santini *et al.*, 1989). Patients having been treated between January 1988 and January 1990 with plasma 5-FU pharmacokinetic data were included in the study. One hundred and eighty-seven patients (155 males, 32 females) with histologically proven and previously untreated head and neck cancer were entered in the study. Only treatment cycles where hepatic function tests and serum protein concentrations were determined (see below) were included in the analysis. In the majority of patients, only the first cycle of 5-FU therapy was analysed. The interval between cycles was 3 weeks. Three successive cycles of therapy were planned for each patient. The median age of the patients was 62.0-years-old (range 35–85). Hematologic tests (complete blood count), hepatic function tests (AST, ALT, alkaline phosphatase, GGT, LDH, and bilirubin), and renal function tests

Correspondence: G. Milano, Laboratoire d'Oncopharmacologie, Centre Antoine-Lacassagne, 36 voie Romaine, 06054 Nice, France.
*Current address: Comprehensive Cancer Center of Wake Forest University, Bowman Gray School of Medicine Winston-Salem, North Carolina, USA.
Received 29 November 1991; and in revised form 1 March 1992

were performed prior to receiving therapy and during the treatment course. Serum proteins (albumin, transferrin, and prealbumin) were also determined and used as markers of nutritional status. Hematologic tests, hepatic function tests, and serum protein concentrations were determined within 1 week (typically the day prior) of the initiation of cytotoxic chemotherapy. On day 1, all patients received cisplatin 100 mg m$^{-2}$ by intravenous infusion followed by 5-FU at starting doses of 550–1069 mg m$^{-2}$ day$^{-1}$ (mean = 875.5 mg m$^{-2}$ day$^{-1}$) on days 2–6 administered by controlled flow pump. Starting doses varied as a function of patients age and performance status. 5-FU doses were routinely adjusted at midcycle (according to the midcycle AUC) to achieve a targeted systemic exposure (Santini *et al.*, 1989). A total of 222 cycles were available for this multiparametric analysis.

*Sample collection and assay methodology*

Blood samples (5 ml) were collected during 5-FU administration at 08:00 and 17:00 h. The samples were immediately centrifuged (10 min, 2500 r.p.m.) and the plasma stored at − 20°C until analysed. Plasma 5-FU concentrations were determined by a HPLC method (Christophidis *et al.*, 1979). The limit of sensitivity for 5-FU was 10 ng ml$^{-1}$. The intra- and interday coefficients of variation for 5-FU were $<$10%.

*Pharmacokinetic analysis*

The 5-FU AUC was determined by the logarithmic trapezoidal method from 0 to 48 h (midcycle) (Yeh & Kwan, 1978). Systemic clearance was calculated by dividing the total dose administered during 48 h by the AUC (0–48 h) (Gibaldi & Perrier, 1982).

*Statistical analysis*

The normality of all pharmacokinetic and biochemical parameters was assessed by Chi-square analysis. For normally distributed data, the mean ± standard deviation (s.d.) is reported. Data not being normally distributed was transformed to the logarithm of its respective value. Relationships between various biochemical parameters (hepatic function tests and serum proteins) and 5-FU clearance were assessed by both simple linear and stepwise, multiple regression analysis. The a priori level of significance was set at $P<0.05$.

### Results

A total of 187 patients (222 cycles) were analysed. Significant interpatient variability for various 5-FU pharmacokinetic parameters was observed (Table I), including an almost ten-fold range in 5-FU clearance. The frequency distribution for 5-FU clearance (222 cycles) is shown in Figure 1. There was a significant relationship between 5-FU dose and the AUC 0–48 h ($r = 0.4433$, $P<0.00001$). Conversely 5-FU clearance (i.e. log clearance) was not modified by 5-FU dose ($r = -0.1034$, $P = 0.124$, Figure 2) suggesting that at the doses evaluated there was no evidence of diminished 5-FU clearance with increased doses.

Significant interpatient variability in hepatic function and plasma nutritional protein concentrations was observed in our patient population (Table II). The results from simple linear regression analysis of various hepatic function tests versus log 5-FU clearance are shown in Table III. Poor linear correlations between log 5-FU clearance and hepatic function tests were observed. The log 5-FU clearance was poorly correlated with the serum concentration of various proteins which served as markers of nutritional status (Table IV). Attempts at correlating several hepatic or nutritional parameters with the variability of log 5-FU clearance by stepwise, multiple regression analysis were unsuccessful in identifying a statistically significant linear model.

### Discussion

Considering the specific objectives of the present study, we attempted to limit potential sources of uncontrolled interpatient variability by including patients with the same localisation of disease, all patients being previously untreated, and all patients receiving the same chemotherapy treatment pro-

**Table I** Summary of pharmacokinetic parameters

| *Parameter* | *Mean ± s.d.* | *Range* |
|---|---|---|
| Dose (mg m$^{-2}$) | 875.5 ± 136.6 | 552–1069 |
| 5-FU $AUC_{0-48\,h}$ (ng ml$^{-1}$ × h) | 11921.7 ± 5325.4 | 3600–41095 |
| 5-FU Cl (ml min$^{-1}$ m$^{-2}$) | 2820.7 ± 1053.3 | 791–7769 |

**Figure 1** Histogram distribution of 5-FU clearance in the studied population.

**Figure 2** Scattergram for the evolution of log 5-FU clearance as a function of the dose.

**Table II** Summary of laboratory parameters

| *Parameter*[a] | *Mean* ± s.d. | Range |
|---|---|---|
| Serum creatinine (<12 mg l$^{-1}$) | 9.5 ± 2.5 | 5.5–21 |
| AST (<25 IU l$^{-1}$) | 17.5 ± 10.9 | 5–86 |
| ALT (<30 IU l$^{-1}$) | 15.9 ± 16.1 | 4–189 |
| Alkaline phosphatase (<210 IU l$^{-1}$) | 144.5 ± 103.2 | 15–1068 |
| GGT (<37 IU l$^{-1}$) | 52.8 ± 76.1 | 4–491 |
| LDH (<330 IU l$^{-1}$) | 228.4 ± 93.0 | 80–1190 |
| Bilirubin (<17 mg l$^{-1}$) | 6.7 ± 3.5 | 2–28 |
| Albumin (32–45 g l$^{-1}$) | 38.2 ± 6.0 | 18–55 |
| Prealbumin (0.17–0.40 g dl$^{-1}$) | 0.25 ± 0.07 | 0.04–0.53 |
| Transferrin (2.0–4.0 g dl$^{-1}$) | 2.76 ± 0.56 | 1.59–4.39 |

[a]Values in parentheses note normal values.

**Table III** Results of simple linear regression analysis of hepatic parameters versus log 5-FU Cl

| *Parameter* | *r* | *P* |
|---|---|---|
| Log AST | – 0.0526 | 0.4365 |
| Log ALT | – 0.1167 | 0.0842 |
| Log Alk. Phosp. | 0.154 | 0.0214 |
| Log GGT | 0.0652 | 0.3436 |
| Log LDH | – 0.0984 | 0.1563 |
| Log Bilirubin | – 0.1278 | 0.0601 |

**Table IV** Results of simple linear regression of various plasma nutritional proteins versus log 5-FU Cl

| *Parameter* | *r* | *P* |
|---|---|---|
| Albumin | 0.0110 | 0.8714 |
| Prealbumin | – 0.1067 | 0.1129 |
| Transferrin | 0.0439 | 0.5226 |

tocol. The pharmacokinetic parameters observed in our large set of patients are similar to those reported by other investigators following continuous intravenous infusion of 5-FU (Floyd *et al.*, 1982; Thyss *et al.*, 1986*a*; Erlichman *et al.*, 1986). The median value for 5-FU clearance (2664 ml min$^{-1}$ m$^{-2}$) was significantly greater than liver blood flow suggesting that catabolism in other tissues contributes significantly to 5-FU degradation. In this studied population, there were patients with particularly high mid-cycle AUC (up to 41095 mg ml$^{-1}$ × h) being at high risk of toxicity and necessitating dose reduction (Santini *et al.*, 1989). As reported previously, there was a significant relationship noted between the 5-FU dose and AUC (Milano *et al.*, 1988). Several studies have reported the pharmacokinetics of 5-FU to be nonlinear with increasing 5-FU dose (Christophidis *et al.*, 1978; McDermott *et al.*, 1982; Collins *et al.*, 1980). As reported by others (Erlichman *et al.*, 1986), we found the pharmacokinetics of 5-FU following continuous infusion to be linear and clearance to be unchanged with increased 5-FU doses within a 2 fold range (550–1069 mg m$^{-2}$ day$^{-1}$ × 5). This has practical consequences since this observation implies that dose modification of 5-FU administered by continuous intravenous results in proportional changes in total body drug exposure.

Since 5-FU is believed to be extensively catabolised by the liver, it is of concern whether 5-FU doses should be reduced in the presence of hepatic dysfunction. Christophidis and coworkers (1978) were unable to relate bioavailability of 5-FU to various hepatic function tests or to the presence of hepatic metastases. Floyd and associates (1982) reported the clearance of 5-FU to be altered in the presence of hepatic metastases although other studies (Nowakowska-Dulawa, 1990) have found no effect of liver metastases on 5-FU disposition. Traditional tests of hepatic function may not reflect the drug metabolic capabilities of the liver. To assess hepatic oxidative function, investigators have utilised drugs such as antipyrine. Due to the heterogeneity of the cytochrome P-450 system, antipyrine only provides a qualitative reflection of hepatic oxidative function (Vesell, 1991). Since DPD is primarily a cytosolic rather than a microsomal enzyme, it is unlikely that markers such as antipyrine would be useful in predicting 5-FU clearance. We have recently reported a significant relationship (Fleming *et al.*, 1991) between DPD activity in mononuclear cells and 5-FU clearance ($r = 0.716$, $P < 0.0001$). Despite its potential utility in patients with normal organ function, knowledge of lymphocyte DPD activity is probably not of benefit in patients with severe hepatic dysfunction. Our population of patients was well suited for evaluating the influence of hepatic dysfunction of 5-FU as observed by the wide variability of hepatic function tests. However serum bilirubin was not significantly elevated in the majority of our patients (upper range 28 mg $l^{-1}$). Given that the majority of our patients had mild to moderate hepatic dysfunction, additional studies in patients with severe dysfunction are necessary. We observed poor correlations between various hepatic function tests and 5-FU clearance. Since only 12% of patients with head and neck cancer have distant metastases to organs (Merino *et al.*, 1977) including the liver, our population may not reflect what may occur in other populations where liver metastases is more common (digestive cancer, breast cancer). Because pharmacodynamic endpoints (mucositis, leukopenia) were not compared between patients with normal liver function and hepatic dysfunction, we consider that additional studies are necessary to determine if the severity of 5-FU-induced toxicities is different in patients with normal and abnormal hepatic function. There were 21 patients followed for more than one cycle of 5-FU (18 patients with cycle 1 and cycle 2, 3 patients with cycle 1, cycle 2 and cycle 3). In examination of the data for these 21 patients, it did not appear that 5-FU clearance consistently increased or decreased from cycle to cycle (NS, Wilcoxon signed rank test); considering the various hepatic function tests there was a significant reduction in the levels of GGT, alkaline phosphatase and ALT; for the other hepatic function tests there were no significant changes (Wilcoxon signed rank test).

Nutritional status, especially in malnourished patients can influence the metabolism of drugs (Krishnaswamy, 1978), including the disposition of anticancer drugs (Mihranian *et al.*, 1984). A recent study (Davis *et al.*, 1990) reported that rats with protein-calorie malnutrition had significantly decreased 5-FU clearance as compared to rats with normal nutritional status. Our population (head and neck cancer patients) was well suited to evaluate the effect of nutritional status on the disposition of 5-FU. We observed a wide range of nutritional status as reflected by the wide dispersion of visceral protein data. Visceral proteins thought to be most relevant for evaluating nutritional status include albumin, transferrin, and prealbumin (Teasley, 1989). We have previously demonstrated serum prealbumin to reflect the nutritional status of cancer patients (Milano *et al.*, 1978). Based on the findings in our study, nutritional status does not influence the clearance of 5-FU. Since toxicities (mucositis, leukopenia) were not assessed in our study, additional studies are needed to determine if malnourished patients for other reasons than pharmacokinetic considerations may have significantly increased toxicity following 5-FU administration as compared to patients of normal nutritional status.

In conclusion, we found 5-FU clearance to be unaffected by dose, hepatic function, or nutritional status. Laboratory data including indices of hepatic function and nutritional status does not appear useful in identifying individuals at risk for altered 5-FU clearance. From a practical point of view and based on pharmacokinetic considerations, dose reductions of 5-FU are not indicated in the presence of mild to moderate hepatic dysfunction or altered nutritional status.

Supported by: French Federation of Cancer Centers and Ligue Nationale Française Contre le Cancer. Dr R. Fleming is a recipient of a Chateaubriand Scholarship from the Scientific Mission, French Embassy, U.S.A.

### References

AMREIN, P.C. & WEITZMAN, S.A. (1985). Treatment of squamous-cell carcinoma of the head and neck with cisplatin and fluorouracil. *J. Clin. Oncol.*, **3**, 1632.

AU, J.L., RUSTUM, Y.M., LEDESMA, E.J. *et al.* (1982). Clinical pharmacological studies of concurrent infusion of 5-fluorouracil and thymidine in treatment of colorectal carcinoma. *Cancer Res.*, **42**, 2930.

CHRISTOPHIDIS, N., VAJDA, F.J., LUCAS, I. *et al.* (1978). Fluorouracil therapy in patients with carcinoma of the large bowel: a pharmacokinetic comparison of various rates and routes of administration. *Clin. Pharmacokin.*, **3**, 330.

CHRISTOPHIDIS, N., MIHALY, G., VADJA, F. & LOUIS, W. (1979). Comparison of liquid-and gas-liquid chromatographic assays of 5-fluorouracil in plasma. *Clin. Chem.*, **25**, 83.

COLLINS, J.L., DEDRICK, R.L., KING, F.G., SPEYER, J.L. & MYERS, C.E. (1980). Nonlinear pharmacokinetic models for 5-fluorouracil in man: intravenous and intraperitoneal routes. *Clin. Pharmacol. Ther.*, **28**, 235.

DAVIS, L.E., SHINKWIN, M.A., SHOU, J. *et al.* (1990). Decreased 5-fluorouracil clearance in protein-calorie malnutrition. *Clin. Pharmacol. Ther.*, **146**, (abstr).

DIASIO, R.B. & HARRIS, B.E. (1989). Clinical pharmacology of 5-fluorouracil. *Clin. Pharmacokin.*, **16**, 215.

ENSMINGER, W.D., ROSOWSKY, A., RASO, V. *et al.* (1978). A clinical-pharmacological evaluation of hepatic arterial infusions of 5-fluoro-2′-deoxyuridine and 5-fluorouracil. *Cancer Res.*, **38**, 3784.

ERLICHMAN, C., FINE, S. & ELHAKIAN, T. (1986). Plasma pharmacokinetics of 5-FU given by continuous infusion with allopurinol. *Cancer Treat. Rep.*, **70**, 903.

FLEMING, R.A., MILANO, G., THYSS, A., RENÉE, N., SCHNEIDER, M. & DEMARD, F. (1991). Correlation between dihydropyrimidine dehydrogenase activity in peripheral mononuclear cells and 5-FU clearance in cancer patients. *Cancer Res.*, **52**, 2899.

FLOYD, R.A., HORNBECK, C.L., BYFIELD, J.E. *et al.* (1982). Clearance of continuously infused 5-Fluorouracil in adults having lung or gastrointestinal carcinoma with or without hepatic metastases. *Drug Intell Clin. Pharm.*, **16**, 665.

GIBALDI, M. & PERRIER, D. (1982). *Pharmacokinetics* (eds). New York: Dekker.

GOLDBERG, J.A., KERR, D.J., WILLMOTT, N. *et al.* (1988). Pharmacokinetics and pharmacodynamics of locoreginal 5-fluorouracil (5-FU) in advanced colorectal liver metastases. *Br. J. Cancer*, **57**, 186.

GREM, J.L. (1990). Fluorinated pyrimidines. In Chabner, C.A., Collins, J.M., (eds) *Cancer Chemotherapy. Principles and Practice.* Philadelphia: Lippincott, 180.

KISH, J., DRELICHMAN, A., JACOBS, J., HOSCHNER, J., KINZLE, J., LOH, J. *et al.* (1982). Clinical trial of cisplatin and 5-FU infusion as initial treatment for advanced squamousa cell carcinoma of the head and neck. *Cancer Treat. Rep.*, **66**, 471.

KRISHNASWAMY, K. (1978). Drug metabolism and pharmacokinetics in malnutrition. *Clin. Pharmacokin.*, **3**, 216.

McDERMOTT, B.J., VAN DER BERG, H.W. & MURPHY, R.F. (1982). Nonlinear pharmacokinetics for the elimination of 5-fluorouracil after intravenous adminstration in cancer patients. *Cancer Chemother. Pharmacol.*, **9**, 173.

MERINO, O.R., LINDBERG, R.D. & FLETCHER, G.H. (1977). An analysis of distant metastases from squamous cell carcinoma of the upper respiratory and digestive tracts. *Cancer*, **40**, 145.

MIHRANIAN, M.H., WANG, Y.M. & DALY, J.M. (1984). Effects of nutritional depletion and repletion on plasma methotrexate pharmacokinetics. *Cancer*, **54**, 2268.

MILANO, G., COOPER, E.H., GOLIGHER, J.C., GILES, G.R. & NEVILLE, A.M. (1978). Serum prealbumin, retinol-binding protein, transferrin and albumin levels in patients with large bowel cancer. *J. Natl Cancer Inst.*, **61**, 687.

MILANO, G., ROMAN, P., KHATER, R., FRENAY, M., RENÉE, N. & NAMER, M. (1988). Dose versus pharmacokinetics for predicting tolerance to 5-day continuous infusion of 5-FU. *Int. J. Cancer*, **41**, 537.

NOWAKOWSKA-DULAWA, E. (1990). Circadian rhythm of 5-fluorouracil (fu) pharmacokinetics and tolerance. *Chronobiologia*, **17**, 27.

SANTINI, J., MILANO, G., THYSS, A. *et al.* (1989). 5-FU therapeutic monitoring with dose adjustment leads to improved therapeutic index in head and neck cancer. *Br. J. Cancer*, **59**, 287.

TEASLEY, K.M. (1989). Assessment, prevalence, and clinical significance of malnutrition. In DiPiro, J.T., Talbert, R.L., Hayes, P.E., Yee, G.C. & Posey, L.M. (eds) *Pharmacology: a Pathophysiologic Approach*. New York: Elsevier, 1571.

THYSS, A., MILANO, G., RENÉE, N. *et al.* (1986*a*). Clinical Pharmacokinetic study of 5-FU in continuous 5-day infusions for head and neck cancer. *Cancer Chemother. Pharmacol.*, **16**, 64.

THYSS, A., SCHNEIDER, M., SANTINI, J., CALDANI, C., VALLICIONI, J., CHAUVEL, P. *et al.* (1986*b*). Induction chemotherapy with cis-platinum and 5-fluorouracil. *Br. J. Cancer*, **54**, 755.

TRUMP, D.L., EGORIN, M.J., FORREST, A. *et al.* (1991). Pharmacokinetic and pharmacodynamic analysis of 5-fluorouracil during 72 hours continuous infusion with and without dipyridamole. *J. Clin. Oncol.* (in press).

VESELL, E.J. (1991). The model drug approach in clinical pharmacology. *Clin. Pharmacol. Ther.*, **50**, 239.

YEH, K.C. & KWAN, K.C. (1978). A comparison of numerical algorithms by trapezoidal, LaGrange, and spilne approximations. *J. Pharmacokin. Biopharm.*, **6**, 79.